# The Most Likely Time and Place of Introduction of BTV8 into Belgian Ruminants

**DOI:** 10.1371/journal.pone.0009405

**Published:** 2010-02-24

**Authors:** Claude Saegerman, Philip Mellor, Aude Uyttenhoef, Jean-Baptiste Hanon, Nathalie Kirschvink, Eric Haubruge, Pierre Delcroix, Jean-Yves Houtain, Philippe Pourquier, Frank Vandenbussche, Bart Verheyden, Kris De Clercq, Guy Czaplicki

**Affiliations:** 1 Department of Infectious and Parasitic Diseases, Faculty of Veterinary Medicine, University of Liège, Liège, Belgium; 2 Department of Arbovirology, Institute for Animal Health, Woking, United Kingdom; 3 Veterinary Department, University of Namur, Namur, Belgium; 4 Department of Functional and Evolutionary Entomology, University of Liège, Gembloux, Belgium; 5 Federal Agency for the Safety of the Food Chain, Brussels, Belgium; 6 Association Régionale de Santé et d'Identification Animales, Loncin, Belgium; 7 ID VET, Montpellier, France; 8 Department of Virology, Veterinary and Agrochemical Research Center, Brussels, Belgium; St. Petersburg Pasteur Institute, Russian Federation

## Abstract

**Background:**

In northern Europe, bluetongue (BT) caused by the BT virus (BTV), serotype 8, was first notified in August 2006 and numerous ruminant herds were affected in 2007 and 2008. However, the origin and the time and place of the original introduction have not yet been determined.

**Methods and Principal Findings:**

Four retrospective epidemiological surveys have been performed to enable determination of the initial spatiotemporal occurrence of this emerging disease in southern Belgium: investigations of the first recorded outbreaks near to the disease epicenter; a large anonymous, random postal survey of cattle herds and sheep flocks; a random historical milk tank survey of samples tested with an indirect ELISA and a follow-up survey of non-specific health indicators. The original introduction of BTV into the region probably occurred during spring 2006 near to the National Park of Hautes Fagnes and Eifel when *Culicoides* become active.

**Conclusions/Significance:**

The determination of the most likely time and place of introduction of BTV8 into a country is of paramount importance to enhance awareness and understanding and, to improve modeling of vector-borne emerging infectious diseases.

## Introduction

Bluetongue (BT) is an infectious but non contagious viral disease caused by bluetongue virus (BTV). BTV belongs to the family *Reoviridae*, genus *Orbivirus* and exists as 24 serotypes [1].

Firstly notified at 17 August 2006, BTV-8, thought to be of possible sub-Saharan origin, initiated an epidemic of BT in northern Europe (mainly The Netherlands, Belgium, and Germany) [2]–[5]. In 2007, following a brief winter halt to its transmission, the virus re-emerged after overwintering via an unidentified mechanism in the previously infected areas [2]. In contrast to 2006 when the virus was identified on some 2000 holdings, more than 40,000 of ruminant holdings became affected in 2007 with many infected animals exhibiting disease (*Animal Disease Notification System*, http://ec.europa.eu/food/animal/diseases/adns/index_en.htm). Indeed, in 2007, the BTV8 expanded its range to include several European countries, involving at the end of the period: Belgium, Czech Republic, Denmark, France, Germany, Luxemburg, the Netherlands, Switzerland and United Kingdom [4]. The great majority of the outbreaks was detected in four countries: France, Germany, Belgium and the Netherlands. Whilst the full animal welfare and economic costs have not yet been fully quantified at the European level but some national studies are available such as the financial consequences of the Dutch bluetongue serotype 8 epidemics in 2006 and 2007 [6].

Currently, introduction of BTV from one area to another is thought to be able to occur in four ways. The first is through movement of viraemic animals (domestic and wild ruminants) or animal germplasm (semen, embryos). The second is by infected vector *Culicoides* carried by various living (plants, animals) or inanimate (airplanes, ships) means. The third is through the active flight of infected vector *Culicoides* (local propagation) and the fourth is through passive flight of infected vector *Culicoides* by the wind (responsible for long-distance dissemination) [4].

In northern Europe in 2006, statistical analysis based on 79.2% of first outbreaks notified before 15 September 2006, showed that the first significant disease cluster (epicentre) was located in The Netherlands, south of Maastricht (border area with Belgium and Germany) and had a 20 km radius [7]. This preliminary investigation was confirmed by a seroprevalence survey of BTV-8 in cattle in the Netherlands in spring 2007 [8] and was supported by Belgian findings [5], [9]. In addition, most evidence of the emerging disease was detected clinically, in the first instance, by veterinary practitioners. While clinical surveillance underestimated the true impact of the epidemic (lack of sensitivity) it indicated the correct spatial trend [9].

Few and limited data concerning the date of real introduction of BTV-8 in the northern European epicentre are currently published. The presumptive earliest date when clinical signs were observed was on the 30–31 July 2006 in The Netherlands [10]. Retrospective preliminary reports on the first observed BTV outbreaks in Belgium and Germany indicate that the first BTV clinical signs appeared around 17 July to 5 August 2006 ([11], [12]). In late June, Belgian veterinarians saw an unusual number of bovine cases that they primarily attributed to photosensitization or exposure to mycotoxins (sporidesmins), entities that could be included in the differential diagnosis of BT [13], [14]. Moreover, a longitudinal study of clinical BT cases in cattle indicated that photosensitization-like lesions may occur at a late stage in BT suspected and subsequently confirmed cases [15].

In the past, several retrospective and proactive studies have been successfully conducted to determine the first occurrence of an emerging infectious disease (EID) in a country (e.g., transmissible spongiform encephalopathies and bovine parafiliariosis) [16], [17] but limited studies have been carried out on the incursion of BTV-8 into northern Europe (e.g., [18]). Possible routes of BTV-8 introduction into the original epicentre of the epidemic in northern Europe were investigated from 1 January 2006 through 18 August 2006 but the exact route of the introduction remained unknown [19]. However, the choice of this starting date implies introduction of BTV-8 in 2006.

The aim of the current investigation is to provide a first evidence-based study on the most likely time and place of introduction of BTV-8 into southern Belgium. To this effect, four epidemiological surveys were conducted near to the Belgian epicentre of the BT epidemic and in the surrounding area.

## Materials and Methods

### Retrospective Survey near to the Epicentre of the 2006 Epidemic

The first 87 BT notified outbreaks confirmed by RTqPCR and/or competitive ELISA and identified in the province of Liège (near to the epicentre of the BT epidemic) during the period from 17 August 2006 through to 1 November 2006 were investigated. Three parameters were followed. The first was the distribution time of the first putative clinical appearance of BT (attributed to the BT by the farmers). The second was the duration between the first clinical appearance of BT and the first outbreak notification to the authorities and the third was the spatio-temporal distribution of quantification cycle (Cq) values of the RTqPCRs performed in cattle during the two week periods after the first BT notifications (*N* = 32). A Cq value corresponds to the fractional PCR cycle at which the target is quantified. Cq is inversely proportional to the amount of gene copies present in the sample and its value, for each sample, was used to compare the blood viral load [20].

### Anonymous, Random Postal Surveys in Southern Belgium

During the winter 2006–2007, a BT awareness campaign was conducted by the authors. In October 2007, the first anonymous postal survey was sent to sheep and goat breeders who were members of an inter-professional federation in southern Belgium (FICOW for *Filière Interprofessionnelle Caprine et Ovine Wallonne*; *N* = 493). In November 2007, the same anonymous survey was sent to 4,745 cattle breeders randomly selected from livestock keepers in southern Belgium (Table 1). The main objectives of the two parts of the survey were to appraise livestock keepers of the clinical presentation and the economic consequences of BT on farms (not shown in this paper) and also to obtain from farmers estimations of the dates when livestock were seasonally housed and put out on pasture in 2006, the condition of infected and recovered BT-affected animals and the morbidity, mortality and case fatality rates. Response rates of the first and second parts of the survey were 18 and 32%, respectively and were considered as acceptable [21]. Moreover, the spatial (provincial) representation of the responders in comparison with the selected breeders was assessed and considered as good (sheep survey: Pearson correlation coefficient = 0.87, *P* = 0.02; cattle survey: Pearson coefficient = 0.96, *P* = 0.01). The difference in the response rate between the two parts of the survey was attributed to a more structuring of the cattle than the sheep livestock (more hobby sheep breeders).

**Table 1:** Design of the retrospective surveys.

Province	Sheep postal survey (University of Liège and University of Namur)		At random cattle postal survey (University of Liège)			At random milk tank survey (ARSIA)		
	Members	Responded	Total herds	Contacted	Responded	Dairy herds	Contacted	Responded
Brabant	42	9	675	198	61	184	23	7
Hainaut	107	26	4,101	1,417	449	1,740	182	67
Liège	134	26	3,678	998	280	1,650	178	57
Luxembourg	90	9	2,926	1,145	437	756	95	37
Namur	120	19	2,480	987	273	756	78	38
Other	8	3	-	-	-	-	-	-
Total	493	89	13,860	4,745	1,500	5,086	556	206

### Random, Historical, Milk Tank Survey in Southern Belgium

**Figure 1:**
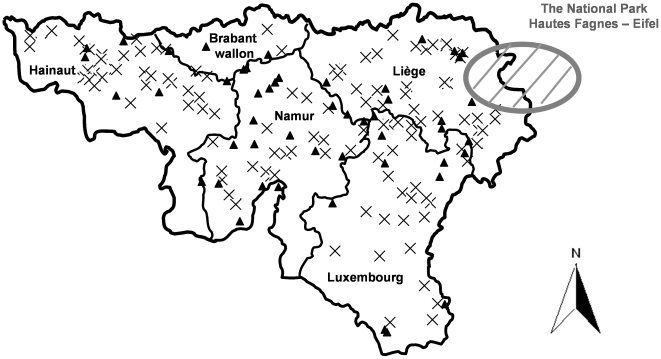
Study area and random milk tank survey. Southern Belgium with its 5 provinces and location of 206 randomly selected dairy herds tested for BTV antibodies on milk tank samples (black cross, the 156 dairy herds tested negative during the period between 12^th^ January 2006 and 14^th^ March 2006; black triangle, the 50 dairy herds tested negative during the previous period but tested positive during the period between 6^th^ December 2007 and 3^rd^ February 2008).

5,086 dairy herds are located in southern Belgium. A voluntary leptospirosis survey was conducted on a sub-set of these farms by ARSIA in the beginning of 2006 which involved random sampling of 566 dairy herds. In this survey, herd holders were asked to complete an epidemiological questionnaire containing several herd level prediction variables (farm demographics, management practices and observed clinical signs during the previous 12 months, for calves, heifers and cows respectively) and to submit a sample of bulk milk from the holding for testing. 206 dairy farms responded during the period 12 February 2006 through to 14 March 2006. The responders were statistically representative of the farms surveyed (Pearson correlation coefficient = 0.97, *P* = 0.006). The bulk milk samples were centrifuged and stored at −20°C and were retrospectively checked for antibodies against BTV. From 6 December 2007 through to 3 February 2008, a second bulk milk sample was taken for BTV analysis from 50 (random selection) of the 206 previously tested dairy farms (Figure 1).

### Databases

Databases of BT notification from the Federal Agency for Safety of the Food Chain (Brussels, Belgium) and concerning milk production indicators from the Association Wallonne de l'Elevage (Ciney, Belgium) were consulted. In the milk production database two non specific indicators were monitored, monthly from January 2000 to December 2007: the mean milk yield by lactating cow expressed in kilograms calculated at each milk control period under test (monthly intervals) and the mean cellular score (MCS) with the following formula:
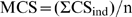
and
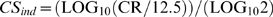
With:

*ind* = Each animal of a milk production unit

*n* = Number of animals in the milk production unit

*CR* = Number of cells obtained during the milk control period under test (varied from 0 through 9999)

*CS_ind_* = varied from 0 to 9, was rounded with one decimal (e.g., 5.4); if *CS_ind_*>9 then *CS_ind_* = 9 and if CS_ind_<0 then CS_ind_ = 0.

Because non increase trend of milk yield was observed in each of the dairy herds followed during the period before the BT emergence, each value obtained monthly from 2006 to 2007 was compared with the mean value obtained during the period from 2000 to 2005 (with the 95% confidence interval [CI]). A drop in milk yield, DMY (value under the lower limit of the 95% CI) and an increase of MCS (value above the upper limit of the 95% CI) were linked to non specific disease indicators.

### BTV RNA Detection in EDTA-Blood Samples

EDTA-blood samples were collected in some outbreak areas near to the epicentre of the BT epidemic and stored at +4°C before analysis at the VAR-CODA-CERVA. These samples were used to detect the levels of “viraemia” by real-time RT-PCR (RT-qPCR), which detects BTV RNA, according to Vandenbussche et al. [18]. In order to amplify the BTV genome, a RT-qPCR amplifying a region of BTV segment 5 (RT-qPCR_S5) was used [22]. Bovine beta-actin was contemporarily amplified as an internal control [5]. Quantification cycle (Cq) values, for each sample, were determined to estimate the blood viral RNA load [20].

### Bluetongue Virus Antibody ELISA

A competitive BTV antibody ELISA designed for use on serum samples (cELISA) was performed by CERVA-CODA [18] according to manufacturer's instructions (IDVET, Montpellier, France). An indirect BTV antibody ELISA designed for use on bulk milk samples (mELISA) [23] was performed by ARSIA according to manufacturer's instructions (ID.VET, Montpellier, France). Briefly, 50 µl skimmed milk from the holding tank and 50 µl “wash solution” (final sample dilution 1∶2) were added to the wells of a BTV-VP7-coated micro-titre plate. After incubation for 45 min at room temperature (rT), plates were washed and incubated with 100 µl anti-ruminant peroxidase conjugate for 30 min at rT. After washing, wells were incubated for 15 min at rT with 100 µl TMB substrate. Colour development was stopped by the addition of 100 µl 0.5 M H_2_SO_4_. The ratio optical density of the sample/optical density of the positive control (S/P) expressed in percent was calculated using the following formula:
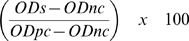
with “*OD*” for optical density values measured at 450 nm, “*s*” for sample; “*nc*” for negative control and “*pc*” for the positive control.

All tests were performed by the same operator and by using plates with a same batch number in order to optimize standardization of the analysis. A S/P% ≤30%, between 30% and 40% or ≥40% was considered as negative, doubtful and positive respectively.

### Statistical Analysis

The spatial representativeness of responders among the breeders who were contacted was statistically evaluated by the Pearson correlation coefficient [24].

Comparisons of morbidity, mortality and case fatality rates between cattle and sheep and between sub-groups of the same species were performed using a two-sample Wilcoxon rank-sum (Mann-Whitney) test and by assuming an unequal variance and non-normal data distribution [24]. A Welch's test was used to compare the spatio-temporal distribution of the Cq values in cattle outbreaks during the two first weeks after the appearance of the BT in province of Liège [24]. The limit of statistical significance of the tests was defined as *P*≤0.05.

### Ethics Statement

No ethics statement was required for the collection of blood and milk samples as well as for the epidemiological surveys described above. Blood samples were obtained from different veterinary practitioners visiting farms with suspected animal according to the request of owners (implementation of the Belgian legislation). Milk samples were obtained from the ARSIA with the permission of the owners. Epidemiological postal surveys were obtained with the help of owners on a voluntary basis.

## Results

### Retrospective Survey Near to the Epicentre of the Epidemic

**Figure 2:**
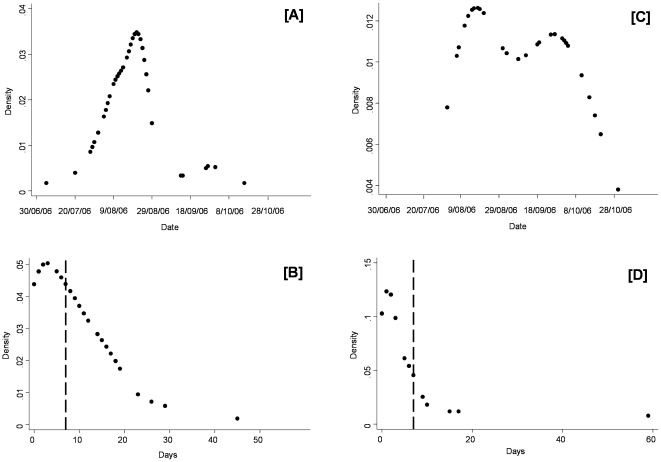
Time of first clinical BT appearance ([A], [C]) and the duration ([B], [D]) between this time and the first outbreak notification to the Authorities (87 first outbreaks in province of Liège). Date: day/month/year; [A] and [B]: 57 cattle outbreaks; [C] and [D]: 30 sheep outbreaks; For [B] and [D], the vertical dotted line corresponds to a duration of 7 days.

Each epidemiological inquiry performed in 87 of the first BT outbreaks (57 cattle herds and 30 sheep flocks) near the epicentre of the epidemic (province of Liège) was investigated. The time distributions of the first clinical appearance of BT in these outbreaks, based on farmer's opinion, are presented in Figure 2 A (for cattle herds) and Figure 2 C (for sheep flocks). The first clinical signs of BT were observed in cattle, but there were two waves of outbreaks in both species but more accentuated for sheep, with a junction point in the beginning of September 2006 (Day 250). For these outbreaks, the duration between the first clinical appearance of BT and the first outbreak notification are presented in Figure 2 B (for cattle herds) and Figure 2 D (for sheep flocks). The trend for both ruminant species is the same but sheep outbreaks were more rapidly notified (83% in the first week) than cattle outbreaks (54% in the first week).

**Figure 3:**
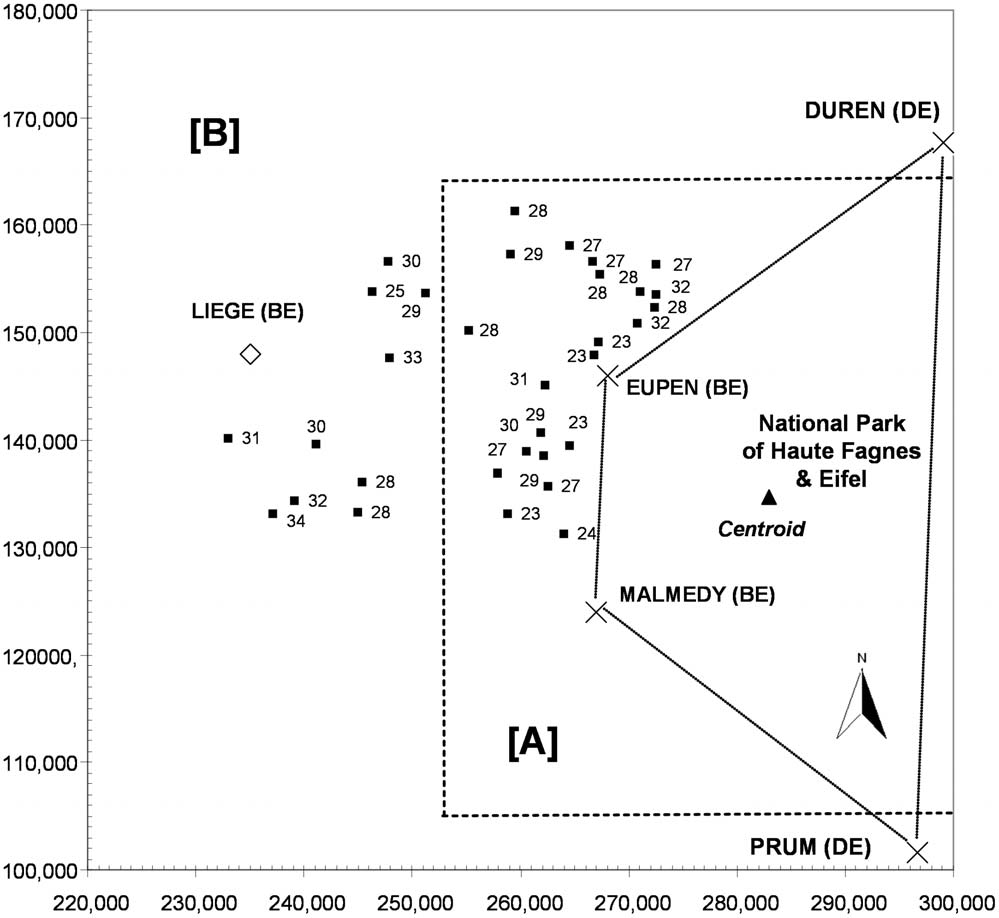
Spatial distribution of Cq values observed in cattle herds in the eastern part of the province of Liège during the first 2 weeks after the appearance of bluetongue. [A] and [B]: Areas close to and remote from the National Park of Hautes Fagnes and Eifel, respectively. The two areas were delimited according to a distance of 30 km from the centroid of the National Haute Fagnes – Eifel. BE = located in Belgium; DE = located in Germany.

For cattle during the two week periods after the first BT notifications, no significant spatial difference between the first clinical appearance of BT and the first outbreak notification was observed in the eastern part of the province of Liège (Welch' test; *P*>0.50); which means the difference is about 10 days in all parts of the area under consideration. In addition no significant temporal difference was observed in the distribution of percentage of inhibition in cELISA and Cq values of the RTqPCR performed on cattle herds during the first 2 weeks after the appearance of BT (Welch' test; *P*>0.44). However a significant spatial gradient was observed in the province of Liège where the mean Cq values increased from the area bordering the National Park of Hautes Fagnes and Eifel (suggesting a higher viraemia) in the east of the province, towards the west (Figure 3). Indeed, the mean of Cq values in the area [A] was significantly lower (higher viraemia) than the [B] (*P* = 0.0065). In addition, the proportion of positive cELISA results (concomitant with Cq ≤33) increased with the same gradient (i.e. close to and remote from the National Park of Hautes Fagnes and Eifel: 19/22 and 8/9, respectively).

### Anonymous Random Postal Survey in Southern Belgium

**Figure 4:**
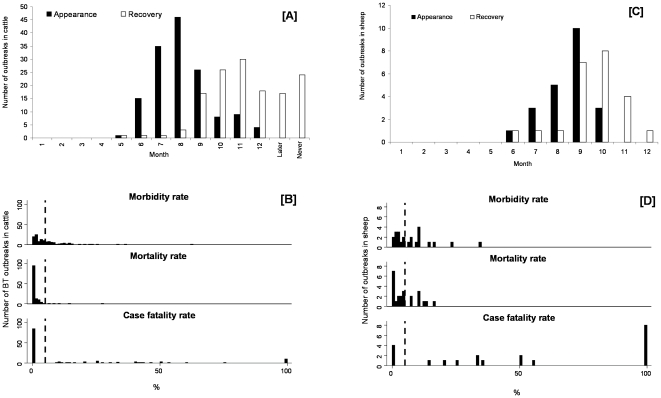
Starting month of clinical appearance and recovery ([A], [C]), and morbidity, mortality and case fatality rates ([B], [D]) for cattle and sheep outbreaks in southern Belgium (random surveys). [A] and [B]: 144 cattle outbreaks confirmed by RTqPCR and/or competitive ELISA; [C] and [D]: 23 sheep outbreaks confirmed by RTqPCR and/or competitive ELISA; Later: if recovery was observed after December 2006; Never: if recovery was not observed before the end of your study (maybe those cows are recovered by now); For [A] and [C], only 2 cattle breeders and 1 sheep breeder observed BT clinical appearance before May 2006 and June 2006, respectively but there is not further information regarding the precise time point so these observations are not cited in the figure; For [B] and [D], the vertical dotted line corresponds to a rate of 5%.

The monthly distribution of the first appearance of clinical signs and the recovery of diseased animals is presented in Figure 4 A and C. In cattle, the first clinical appearance of BT for which we have specific timing was in May 2006 while for sheep it was in June. In addition, 2 cattle breeders and 1 sheep breeder observed clinical BT before May 2006 and June 2006, respectively but more specific timing was not available from these breeders. The rate of increase in the first appearance of disease was relatively similar in both species with a peak in August and September for cattle and sheep respectively. The recovery period of affected animals was much longer for cattle than for sheep.

The distribution of morbidity, mortality and case fatality rates are presented in Figure 4 B and D. In 2006, non-significant differences were observed for morbidity rate between cattle (Median = 3.8%) and sheep (Median = 6.9%) (*P*<0.21). However, the mortality rate was significantly higher in sheep (Median = 3.6%) than in cattle (Median = 0%) (*P*<0.0001). The case fatality rate was also significantly higher in sheep (Median = 50%) than in cattle (Median = 0%) (*P*<0.0001).

### Random Historical Bulk Milk Sampling

All bulk milk samples originating from 206 randomly selected dairy herds in southern Belgium, collected between 12^th^ February 2006 and 14^th^ March 2006 tested negative for the presence of BTV antibodies. Among them, 50 dairy herds underwent a second sampling between 6^th^ December 2007 and 3^rd^ February 2008. All of these samples tested positive for the presence of BTV antibodies. In addition, 9 milk samples were diluted to confirm the presence of high level of antibodies (titres ranged from 32 to 128).

The herd size, the morbidity and mortality rates of this sub-group of dairy herds were compared with other notified outbreaks among the 206 dairy herds (excluding the 50 farms above). No significant differences were observed (Two-sample Wilcoxon rank-sum test, *P*>0.05).

### Follow-Up of Milk Production Indicators

**Figure 5:**
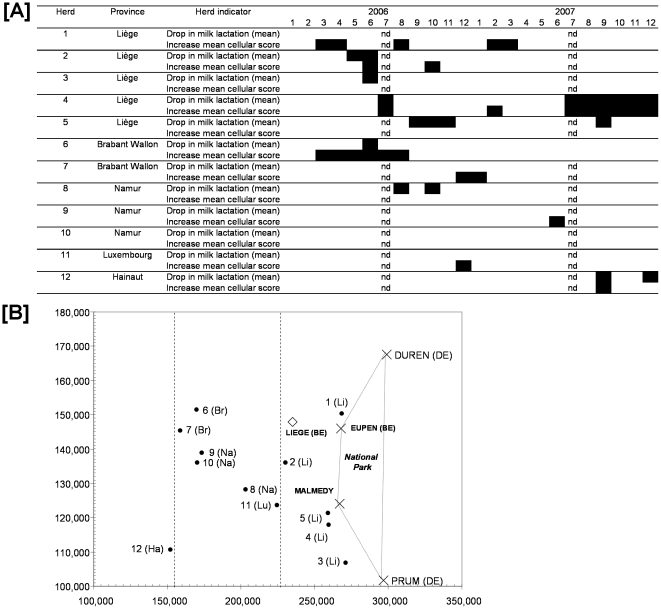
Temporal [A] and spatial [B] distributions of 2 disease non specific indicators at the dairy herd level. DMY: Drop in milk yield (mean by animal and monthly milk control expressed in kilograms); MCS: mean cellular score; ▪: DMY or increase in MCS; nd: Not determined; 1 to 12: Months for 2006 or 2007, respectively; For [B], X axis and Y axis are geographical Lambert coordinates; Li: Province of Liège; Br: Province of Brabant Wallon; Na: Province of Namur; Lu: Province of Luxembourg; Ha: Province of Hainaut; BE: Belgium; DE: Germany.

For 12 of the 50 dairy herds with BTV antibodies in bulk milk samples, a follow-up analysis of milk health production indicators was performed at monthly intervals. The aim was to identify changes in performance and production at herd level, after the first sampling performed between 13^th^ February 2006 and 22 February 2006 that could be linked with the appearance of disease or infection (Figure 5). Considering the spatiotemporal distribution of these indicators, the progression of the disease was probably from the east (province of Liège) to the west as far as the province of Hainaut, and the first indicator changes were observed during the spring of 2006.

## Discussion

In northern Europe the first notification of BT in a sheep occurred on 17^th^ August 2006 in The Netherlands [25] and immediately after a BT case in cattle was confirmed in Belgium. The virus was identified as a strain of BTV-8 of possible sub-Saharan origin [2] which, subsequent to August 2006, was the cause of a severe epizootic of BT in northern Europe [4] with the primary cluster of cases being located around Maastricht (The Netherlands) [26]. The exact route of introduction of BTV-8 into northern Europe remains unknown [19]. However, limited published clinical data, as summarized in this paper, suggest that BTV was present in northern Europe at some time before August 2006. Nevertheless, sera randomly selected from a bovine herpesvirus-1 screening programme carried out in Belgium in 2004 (*N* = 338) and 2005 (*N* = 365) and retrospectively checked for the presence of BTV antibodies were all negative [18], suggesting that BT was absent before 2006 in Belgium. In addition, serum samples from apparently healthy, hunter-killed red deer (*Cervus elaphus*) in southern Belgium collected during the autumns of 2005 (*N* = 200), 2006 (*N* = 469) and 2007 (*N* = 513) were also checked for BTV antibodies. All sera sampled in 2005 were negative but low and high seroconversions were detected in 2006 (0.9% with a 95% CI: 0.2–2.2%) and 2007 (40.4% with a 95% CI: 36.1–44.7%), respectively [27]. In autumn 2006, 4 seropositive red deer were observed in the province of Liege in the surroundings of the National Park of Hautes Fagnes and Eifel (Linden, personal communication). This park is a Belgian - German initiative to protect the Eifel high plateau which straddles the two countries.

Prior to the BT epidemic, data on Palaearctic *Culicoides* species midges in Belgium were only available from a study carried out over the period 11^th^ May–31^st^ December 2006 at the Walloon Agricultural Research Center (CRA-W) at the Gembloux site (50°33′42″N – 4°42′38″E; altitude 170 m; cultivated land within 1 km of livestock) [28]. In this study, the main species reported were (by decreasing order): *C. obsoletus*/*C. scoticus*, *C. chiopterus*, *C. punctatus* and *C. dewulfi*. The two first species have a longer flight period starting in mid April and ending in November [29]. The main flight peak was recorded in June when the average temperature was increasing, at the same time as wind and rainfall were decreasing. In addition, it was shown that the ratio of females/males increased from May to July 2006 and then decreased. In relation to these *Culicoides* species, some have recently been assessed for ability to support BTV-8 replication to transmissible levels subsequent to their oral infection [2]. These authors showed that *C. scoticus* is able to support the replication BTV-8 to high levels, providing thereby the first evidence of the potential of this species to transmit BTV-8.

A spatio-temporal retrospective study on the introduction of BTV into a country is of paramount importance to enhance awareness and understanding and, to improve modeling of vector-borne EID. Using the example of Belgium, the present study has strived to reach this goal.

The first retrospective survey described in this paper (based on farm opinions) suggests that the initial appearance of clinical signs of BT in cattle was in May 2006 (Czaplicki previously related clinical appearance of BT in July 2006, based on veterinary opinions [12]) though when they did occur clinical signs in sheep were notified more rapidly. The first detection of BT in cattle in Belgium may be related to the larger cattle *vs.* sheep populations, the better clinical surveillance of dairy cattle on pasture compared to sheep and also to the fact that cattle are preferred host of vector *Culicoides* and so might be expected to be infected before sheep. In fact cattle appeared to be the most attractive host for Palaeartic biting midges [30]. The more rapid notification of BT outbreaks in sheep, one these commenced, is likely to be linked to the fact that most breeds of improved sheep exhibit more frequently and severe clinical signs of BT than do cattle (lesions in sheep are more oedematous and haemorrhagic than cattle [31]), so infection in sheep is likely to be detected by owners more easily. Moreover, in Belgium, sheep breeders are frequently hobby farmers and the effect of BT restrictions are more easily supportable than professional farmers.

In addition to the above, the spatial distribution of the Cq values recorded by the BT RTqPCR indicated a relatively higher viral (RNA) load in areas near to the National Park of Hautes Fagnes and Eifel (a destination area for many migrating birds from sub-Saharan Africa) and a decrease in viral load from this area to the west, across the eastern part of the province of Liège. According to recent experimental infection in cattle with BTV-8 [32], the kinetics of the Cq values and clinical score were determined. Cq values decreased progressively after infection and had a value of about 28 at 7–15 days post infection (dpi). In addition, the clinical score increased rapidly after about 4 dpi. The decreasing Cq values observed in samples from animals in areas near to the National Park of Hautes Fagnes and Eifel, towards the west, across the eastern part of the province of Liège, is probably linked to a higher viraemia and to earlier infections in the area near to the National Park. It is emphasised that this explanation holds true only in the context of the early stage in the first emergence of the disease and not in the current enzootic situation, which is frequently the reverse. In addition, the proportion of animals positive by RTqPCR (with Cq ≤33), that were also positive by cELISA was lower in the area close to the National Park. This suggests that there may have been an early introduction of BTV into the National Park with movement out of the Park and towards the west, possibly via the flight of infected biting midges.

The results above were obtained by a large anonymous random, postal survey carried out by the Veterinary Faculty (University of Liège, ULg and University of Namur) after a awareness campaign had been conducted to explain the clinic pattern of BTV-8 infection (the first outbreaks were followed by a multidisciplinary team of the ULg). According to this survey, the starting date of pasturing was similar for the two species and occurred in spring 2006 but the housing period tended to start earlier in sheep than in cattle. Because there are more cattle than sheep in southern Belgium and also because vector *Culicoides* prefer to bite cattle, a greater number of cattle than sheep were exposed to vector midges in spring. The earlier start to the housing period for sheep could be a reason for the relatively milder sheep outbreaks observed in autumn. Moreover the presence of sheep in pasture, early in the year, had probably particular ecologic interactions with *Culicoides* species population. The results of this survey also suggest that clinical signs of BT appeared first in cattle, in May 2006, and then appeared in sheep, in June 2006. Thus the real emergence of BT in Belgium probably occurred in spring 2006 when *Culicoides* become active. The morbidity, the mortality and the case fatality rates were extended for the two species. Whilst no significant difference was observed between species for morbidity, mortality and case fatality rates were statistically higher in sheep than in cattle. These observations are further reasons for the more rapid notification of sheep outbreaks.

The random, historical milk tank sampling survey provides evidence that for dairy herds (with daily clinical surveillance) in southern Belgium, including the province of Liège (near to the epicentre), specific BT antibodies were absent from milk at the beginning of 2006. However, a follow-up survey of 50 dairy herds carried out between 6^th^ December 2007 and 3^rd^ February 2008 indicated that by this time all 50 had been infected with BTV. This survey also provides information on the ratio between the numbers of cattle outbreaks that were and were not notified. Indeed a cross check was established between the above 50 BTV positive dairy herds and the BT official status of these farms. It must be stressed that only 20 of the 50 farms were officially notified as BT outbreaks (95% CI [binomial exact]: 26.4–54.8%) and similar discrepancies occurred in all provinces. The low level of notification of infected cattle herds was probably due to the fact that most infected cattle showed no evidence of clinical signs so their infection was not always noticed by farmers and vets.

The non specific follow up of milk production indicators (which should be interpreted with caution because of the limited number of outbreaks and samples) confirmed the previous information about the spatial trend of the BT epidemic in southern Belgium (from the east to the west) and the fact that some changes in indicators (DMY and increase of MCS) were observed in spring 2006, firstly near to the National park of Hautes Fagnes and Eifel. Similar changes were not observed for each herd in previous years – when BTV was not present.

Finally, in Belgium, the original introduction of BTV probably occurred during spring 2006 near to the National Park of Hautes Fagnes and Eifel when *Culicoides* became active.

From our work, some suggestions can be proposed in the event of future cases of EID in the country: first, retrospective studies should be immediately performed which would be facilitated by the setting up and accessing of a serum bank and/or milk tank samples (less costly); second, an awareness campaign should be conducted for the benefit of farmers and veterinarians; third, control measures must be implemented which should be proportionate and flexible; fourth, the development of an early warning system would be valuable especially when it is integrated into a structured epidemiological network (stretching from governmental to farm levels); fifth, targeted surveillance in National Parks such as the Hautes Fagnes and Eifel included a destination area for many migrating birds from sub-Saharan Africa should be recommended and sixth, the early detection of an EID requires the presence and intervention of multidisciplinary teams of trained scientific staff which should be maintained as a national resource. The present study is useful for understanding key factors for EID especially for risk assessment (modeling) of BTV-8 introduction and spread.
